# Influence of intraocular lens subsurface nanoglistenings on functional visual acuity

**DOI:** 10.1371/journal.pone.0173574

**Published:** 2017-03-22

**Authors:** Takahiro Hiraoka, Kazunori Miyata, Takeshi Hayashidera, Masaharu Iida, Keita Takada, Keiichiro Minami, Tetsuro Oshika

**Affiliations:** 1 Department of Ophthalmology, Faculty of Medicine, University of Tsukuba, Ibaraki, Japan; 2 Department of Ophthalmology, Miyata Eye Hospital, Miyazaki, Japan; Universidade do Minho, PORTUGAL

## Abstract

To investigate the influence of intraocular lens subsurface nanoglistenings (SSNGs) on functional visual acuity (FVA), thirty-nine eyes of 29 patients were examined in this study. The SSNG group comprised 19 eyes of 14 patients (75.7± 5.4 years, mean ± standard deviation), and the control group comprised 20 eyes of 15 patients (73.6 ± 6.5 years). The SSNGs were diagnosed on the basis of the typical whitish IOL appearance upon slit-lamp examination and results of densitometry regarding surface light scattering using Scheimpflug images. The FVA measurement system (AS-28; Kowa, Aichi, Japan) was used to examine changes in continuous visual acuity (VA) over time, and visual function parameters such as FVA, visual maintenance ratio (VMR), maximum VA, minimum VA, standard deviation of VA, and number of blinks were assessed. The results were compared between the SSNG and control groups, and correlations of FVA parameters with the intensity of surface light scattering, time after surgery, and age were also evaluated. There were significant differences in VMR (*P* = 0.035) and standard deviation of VAs (*P* = 0.031) between the two groups, although no significant differences were found in baseline VA, FVA, maximum VA, minimum VA, and number of blinks. None of the FVA parameters showed any significant correlations with the intensity of surface light scattering, time after surgery, or age. There is a possibility that VA is unstable during a continuous gazing task in patients with SSNGs.

## Introduction

Over time, particular types of hydrophobic acrylic intraocular lens (IOL) develop a white, opaque appearance because of an increase in light scattering on their surface [1, 2]. Using scanning electron microscopy, Ong et al. [3] observed hydration-related vacuoles up to 120 μm from the surface of these IOLs; the vacuoles have diameters less than 200 nm and are known as subsurface nanoglistenings (SSNGs). The main cause of these phenomena is thought to be water aggregation in the surface layer, which in turn results from phase separation [4, 5]. The intensity of surface light scattering reportedly increases during the years following the IOL implantation [2, 6]; thus, clinicians fear that patients with this particular condition may suffer decreased visual function.

However, results differ as to whether the surface light scattering due to SSNGs degrades visual function. Several studies have suggested that SSNGs have no negative influence on visual acuity (VA) and/or contrast sensitivity [2, 4, 7], whereas various others have now indicated a risk of decline in visual function [6, 8–10]. For instance, Miyata et al. [6] showed that corrected distance VA tended to decrease in patients with surface light scattering of IOLs when the scattering intensity exceeded 50 computer-compatible tape (CCT) steps on a Scheimpflug image. Furthermore, Yoshida et al. [8] reported a patient with decreased VA and contrast sensitivity resulting from excessive surface light scattering in an acrylic IOL; they extracted the IOL and replaced it with a new one, thus improving the degraded visual function. Similarly, Matsushima et al. [9] reported a case series of five patients with decreased vision owing to severe SSNGs and glistening formation in IOLs. The same group also demonstrated that light transmittance was decreased in all the extracted IOLs; they suggested that this had caused the deterioration in visual function [8, 9]. Beheregaray et al. [10] showed that SSNG-associated increases in forward light scattering (FLS) were significantly correlated with a reduction in VA and in contrast sensitivity. The examples given indicate that SSNG-induced surface light scattering affects visual function somewhat, and that this effect depends on the severity of the SSNGs. The impact of these artifacts on quality of vision needs to be further examined from various perspectives.

Visual impairment is usually assessed using conventional VA testing with high-contrast optotypes. Contrast sensitivity and glare testing provide important additional information regarding visual function. However, the results of these examinations are expressed as one value; therefore, they do not reflect the entire spectrum of results. Recently, much attention has been given to the sequential changes in visual function, because daily tasks generally involve continuous, not static, acquisition of visual information. Functional visual acuity (FVA) testing was developed to assess dynamic changes in visual functioning [11–14]; the technique has proven quite useful in detecting masked impairment of visual function in patients with dry eye [11–13, 15, 16], mild cataract [17], posterior capsule opacification after cataract surgery [18], early presbyopia [19], epiretinal membrane [20], and age-related macular degeneration [21]. In addition, FVA testing detected slight visual deterioration induced by viscous eyedrops [22], eye ointment [23], and soft contact lenses (SCLs) [24], which was not identified using conventional VA testing.

Given that many acrylic IOLs have now been implanted, and that SSNG-associated surface light scattering gradually increases over many years [2, 6], it is crucial that investigators evaluate the impact of SSNGs on continuous visual functioning. For this reason, we conducted the current prospective study to investigate the influence of SSNGs on the FVA by comparing the results of the FVA test between eyes with and without SSNGs.

## Subjects and methods

### Participants

We conducted a comparative prospective study at the Miyata Eye Hospital from April 2014 to March 2016. To examine the influence of SSNG on FVA, we established two groups—those with SSNGs and those without—and recruited candidates separately for each group. The eligibility criteria are shown in Table 1. The study was approved by the institutional review board of Miyata Eye Hospital, and adhered to the tenets of the Declaration of Helsinki. After explaining the nature of the study, each patient signed a written consent form before being enrolled. This consent procedure was approved by the IRB.

**Table 1:** Eligibility criteria for SSNG and control groups.

**SSNG group**
1. Patients who underwent uneventful cataract surgery with implantation of a 1-piece or 3-piece AcrySof IOL (Alcon, Inc., Fort Worth, TX) more than 5 years ago
2. Best-corrected visual acuity of 20/25 or better
3. No ocular or neurological diseases that could affect visual acuity (eg, corneal and vitreoretinal disease, uveitis, glaucoma, and other neurological disorders)
4. Absence of posterior capsular opacity and no history of neodymium:YAG laser capsulotomy
5. Existence of typical whitish IOL appearance upon slit-lamp examination by directing the light in 30 or higher degree angle
6. Light scattering in the anterior IOL surface assessed by a Scheimpflug imaging system (EAS-1000; Nidek Co. Ltd., Aichi, Japan) was higher than 50 computer-compatible tapes steps
**Control group**
1. Patients who underwent uneventful cataract surgery with implantation of a 1-piece or 3-piece IOL between 6 months or more and 1 year or less
2. Best-corrected visual acuity of 20/25 or better
3. No ocular or neurological diseases that could affect visual acuity
4. Absence of posterior capsular opacity and no history of neodymium:YAG laser capsulotomy
5. Absence of typical whitish IOL appearance upon slit-lamp examination

SSNG = subsurface nanoglistening, IOL = intraocular lens.

The SSNGs were diagnosed by at least two ophthalmologists on the basis of the typical whitish IOL appearance upon slit-lamp examination. In addition, increased light scattering on the anterior IOL surface was quantitatively evaluated using a Scheimpflug system (EAS-1000; Nidek Co. Ltd., Aichi, Japan). Specifically, we obtained a Scheimpflug slit image of the IOL at the 0-degree meridian and measured the average scattering light intensity of the central 1.00 × 0.25-mm area of the anterior optic surface using the axial densitometry of the computer. The scattering light intensity was expressed in CCT steps ranging from 0 (minimum) to 255 (maximum) [2, 25]. Only eyes with the surface light scattering higher than 50 CCT steps were included in the SSNG group.

### Functional visual acuity measurement system

**Fig 1:**
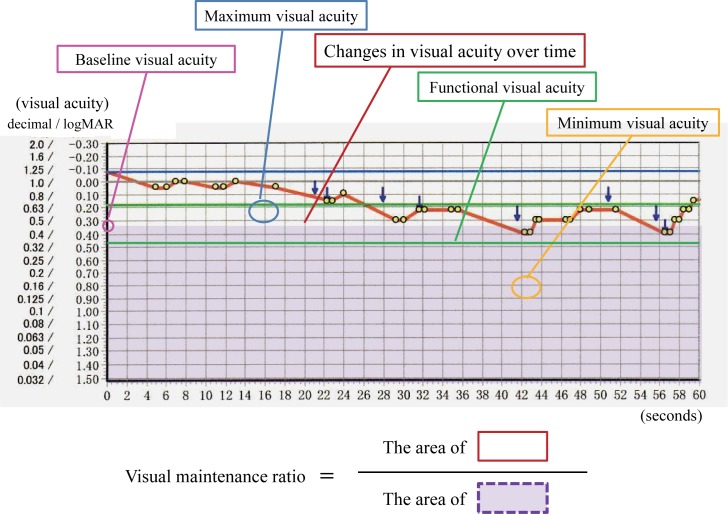
Parameters of functional visual acuity testing. Continuous red line shows sequential visual acuities measured over a 60-second measurement session. Green line denotes functional visual acuity which is calculated as the average of all visual acuity values. Pink circle represents baseline visual acuity. Visual maintenance ratio refers to area beneath time-wise change in visual acuity (red oblique line area) divided by area beneath baseline visual acuity (purple square area). Maximum and minimum visual acuities (blue and orange circles) imply the best and worst values of visual acuity over the testing period.

An FVA measurement system (AS-28; Kowa, Aichi, Japan) was used to examine changes in continuous VA over time. This system has been described in detail elsewhere [11–14]. Briefly, testing was performed monocularly, with best spectacle correction under photopic conditions, and measurements were initiated at the established baseline VA for each patient. Subjects delineated an automatically presented Landolt ring orientation using a joystick. Optotype size was changed in single steps depending on the patient’s responses: the optotype was enlarged when the response was incorrect, and reduced when the response was correct. If there was no response within two seconds, the answer was recorded as an error and the optotype was enlarged. This testing was continuously performed for 60 seconds under spontaneous blinking. The FVA measurements included several evaluation parameters: FVA, visual maintenance ratio (VMR), maximum VA, minimum VA, standard deviation of VAs, and number of blinks. FVA was defined as the average of all visual acuity values measured over time, because this average value may reflect daily vision more efficiently than the visual acuity measured at a specific time point. VMR was defined as FVA divided by baseline VA [13]. Using this index, it was possible to compare groups with different baseline VAs [13, 14]. The standard deviation of VA was used to indicate visual stability (or instability) during the 60-second testing period. Maximum and minimum VAs were defined as the best and worst VAs recorded during the examination, respectively (Fig 1) [26].

### Statistical methods

Regarding the obtained data, normally distributed data were compared between the SSNG and control groups using the Student’s *t*-test, and data that were not normally distributed were compared using the Mann-Whitney U test. In addition, correlations between the various FVA parameters and (1) the intensity of surface light scattering, (2) time after surgery, and (3) age were evaluated using the Pearson correlation test and Spearman correlation test. A *P*-value < 0.05 was considered statistically significant.

## Results

In total, 39 eyes of 29 patients were enrolled in this study. The SSNG group comprised 19 eyes of 14 patients (two men, twelve women) with a mean age of 75.7 ± 5.4 (SD) years (range 66–84 years). The control group comprised 20 eyes of 15 patients (three men, twelve women) with a mean age of 73.6 ± 6.5 years (range 66–88 years). There was no significant difference in age between the groups (*P* = 0.2868). Five different models of the AcrySof IOL (Alcon, Inc., Fort Worth, TX) were used in the implants (MA60BM, MA60AC, MA30BA, SA60AT, and SN60WF). The intensity of surface light scattering was 137.5 ± 43.1 CCT in the SSNG group and 17.0 ± 2.6 CCT in the control group, showing a significant difference (*P* < 0.0001).

Table 2 shows the results of the FVA testing. There were significant differences in VMR (*P* = 0.035) and standard deviation of VAs (*P* = 0.031) between the SSNG and control groups, although no significant differences were found in baseline VA, FVA, maximum VA, minimum VA, and number of blinks. In addition, none of the FVA parameters showed any significant correlations with the intensity of surface light scattering, time after surgery, or age in the SSNG group (Table 3).

**Table 2:** Comparison of FVA testing results between SSNG and control groups.

	SSNG (mean ± SD)	Control (mean ± SD)	*P* value
Baseline VA (logMAR)	-0.08 ± 0.08	-0.07 ± 0.06	0.684
FVA (logMAR)	0.11 ± 0.13	0.04 ± 0.13	0.146
VMR	0.93 ± 0.04	0.96 ± 0.03	0.035*
Maximum VA (logMAR)	-0.06 ± 0.10	-0.08 ± 0.12	0.628
Minimum VA (logMAR)	0.30 ± 0.24	0.18 ± 0.21	0.118
Standard deviation of VA	0.09 ± 0.04	0.06 ± 0.03	0.031†
Number of blinks	13.5 ± 12.6	6.7 ± 5.1	0.088

FVA = functional visual acuity, SSNG = subsurface nanoglistening, VA = visual acuity, logMAR = logarithm of the minimum angle of resolution, VMR = visual maintenance ratio, SD = standard deviation.

*: Significant difference by the Student’s *t*-test.

†: Significant difference by the Mann-Whitney U test.

**Table 3:** Relationship of FVA parameters with the intensity of surface light scattering, time after surgery, and age in the SSNG group.

	Intensity of surface light scattering		Time after surgery (years)		Age (years)	
	Correlation Coefficient	*P* value	Correlation Coefficient	*P* value	Correlation Coefficient	*P* value
Baseline VA (logMAR)	0.358	0.132	0.145	0.591	0.277	0.251
FVA (logMAR)	-0.105	0.668	0.033	0.905	0.346	0.147
VMR	0.353	0.138	0.048	0.861	-0.226	0.352
Maximum VA (logMAR)	0.055	0.822	-0.083	0.760	0.161	0.511
Minimum VA (logMAR)	-0.170	0.487	0.017	0.949	0.378	0.111
Standard deviation of VA	-0.384	0.140	-0.053	0.838	0.113	0.633
Number of blinks	-0.107	0.650	-0.007	0.977	0.106	0.654

VA = visual acuity, logMAR = logarithm of the minimum angle of resolution FVA = functional visual acuity, VMR = visual maintenance ratio.

There were no significant differences in all combinations by the Pearson and Spearman correlation tests.

## Discussion

As shown in the results, the SSNG group had a lower VMR and a larger VA standard deviation than the control group, although the baseline VA did not differ between the groups. The lower VMR implies that patients with SSNGs had difficulty maintaining their baseline VA, and the larger VA standard deviation suggests that the VA fluctuated more widely during the continuous visual task. Together, these findings show that the VA is unstable in eyes with SSNGs. This was the first study to investigate and clarify the influence of SSNG on the stability of vision over time.

In this study, there were no significant correlations between the degree of surface light scattering and visual function parameters, probably because the index of surface light scattering—examined using a Scheimpflug system in this study—reflects backward light scattering (BLS): the light scattered out of the eye towards the light source that can be observed during slit-lamp examinations. In contrast, FLS comprises the light scattered towards the retina, hence it can reduce retinal image contrast, induce glare, and affect visual function [27]. Beheregaray et al. [10] examined both BLS and FLS in patients with SSNGs and showed no association between the two parameters. They also investigated the relationships of BLS and FLS with visual function, finding that increases in FLS, which were evaluated using an optical approach, were significantly correlated with reductions in VA and in contrast sensitivity, whereas BLS was not correlated with visual function in any way [10]. In light of these findings, it stands to reason that there were no significant relationships between the degree of surface light scattering and FVA parameters in this study. Unfortunately, we did not evaluate FLS in the current study; thus, further studies are necessary to clarify the association between FLS and FVA parameters.

We do not have enough data to definitely explain how SSNG-associated surface light scattering causes VA instability; however, several reasons can be assumed. Previous studies revealed that light transmittance is decreased in extracted IOLs with SSNGs [8, 9]. Naturally, lower incident light on the retina reduces visual function. In fact, we previously showed that almost all FVA parameters decreased under low lighting conditions in healthy eyes [28]. Intraocular scattering also degrades retinal image quality [29], so it is unsurprising that patients take longer to judge the orientation of optotypes, even when their decline in VA is not detected using conventional VA testing. The unique feature of the FVA testing system is that a time element is added to VA assessment—sequential measurements are made over a period of time. In addition, the optotype presentation time is ≤ 2 seconds for each measurement; hence, subjects need to rapidly respond to continuously changing optotypes throughout the examination. During conventional VA testing, it often takes time to elicit a response especially in elderly people; however, the acquired result does not reflect this delay in response—only a single VA value is given. In this regard, FVA testing differs from other visual examinations such as conventional VA, contrast sensitivity, and glare disability testing: FVA testing assesses another aspect of visual functioning. This property may be advantageous in that early or small SSNG-induced changes in visual function can be detected. Indeed, this kind of delayed response may also affect reading speed, which was regrettably not examined in the current study. Further studies should be conducted to elucidate this point.

There were some limitations to this study. Firstly, we did not examine FLS. As mentioned, there were no direct associations between the degree of surface light scattering, which corresponds to BLS, and visual parameter degradation in the present study. However, FLS is likely to be more closely related to retinal image quality and visual function [10, 27]. Thus, in future studies, the correlation between FLS and FVA parameters should be investigated in eyes with SSNGs; FLS can be measured using the double-pass or compensation-comparison methods [30–32]. Especially, subjective FLS, which can be evaluated by the latter method through psychophysical straylight measurements, closely predicts functional light scattering [33], and hence it should be examined in relation to visual function in further researches regarding SSNGs.

Secondly, all implanted IOLs were made from hydrophobic acrylic materials by the same company, but light-filtering range (blue-light or ultraviolet-only filtering) and optical design (aspheric or spherical surface) were somewhat different among the IOL models. With regard to light filtering properties, many studies have shown that visual function in eyes with blue-light filtering IOLs is comparable to that in eyes with ultraviolet-only filtering IOLs [34–37]. On the other hand, as for the influence of surface asphericity on visual performance, previous studies showed conflicting results [38–41]. Although several researchers found no difference in visual performance including contrast sensitivity between aspheric and spherical IOLs [38–40], the results of a Meta-Analysis showed that contrast sensitivity in eyes with aspheric IOLs was slightly better than that in eyes with spherical IOLs especially under mesopic conditions, although there was no significant difference in best-corrected visual acuity between the IOLs [41]. In our study, the FVA testing was performed under photopic conditions with high-contrast optotypes, and thus the influence of the difference in IOL asphericity on the study outcomes seems small. In addition, the distribution of the implanted IOL models was similar between the SSNG and control groups (S1 Table). Based on the above, we believe that the study outcomes were not greatly influenced by the difference of IOL models.

Another weakness of our study was the small sample size. Therefore, the current results should be confirmed in a larger study population with a unified IOL model.

In summary, this study investigated the influence of SSNGs on FVA; it found that SSNGs caused instability in VA during a gazing task although they did not seem to induce any decline in standard VA. Furthermore, patients with SSNGs have some difficulty judging or responding quickly to presented optotypes. In the ever-progressing developments of the information society, continuous-gazing tasks, such as reading, work at visual display terminals, and smartphone use are required in many situations of daily life. Driving also requires optimal continuous vision to avoid traffic accidents—especially at night and in bad weather conditions. In addition, the intensity of SSNG surface scattering continuously increases with time [2, 6]. Therefore, the current results need to be corroborated by broader-based studies. Finally, FVA testing detected slight changes in visual function in the current study; this may help physicians identify and understand unknown visual complaints in patients with SSNGs.

## Supporting information

S1 Table Distribution of implanted IOL models in each group. (DOCX)

S1 Dataset (XLS)
