# Correction: Influence of intraocular lens subsurface nanoglistenings on functional visual acuity

**DOI:** 10.1371/journal.pone.0176318

**Published:** 2017-04-17

**Authors:** The PLOS ONE Staff

Fig 1 appears incorrectly in the published article. Please see the correct Fig 1 and its caption here. The publisher apologizes for the error.

**Fig 1:**
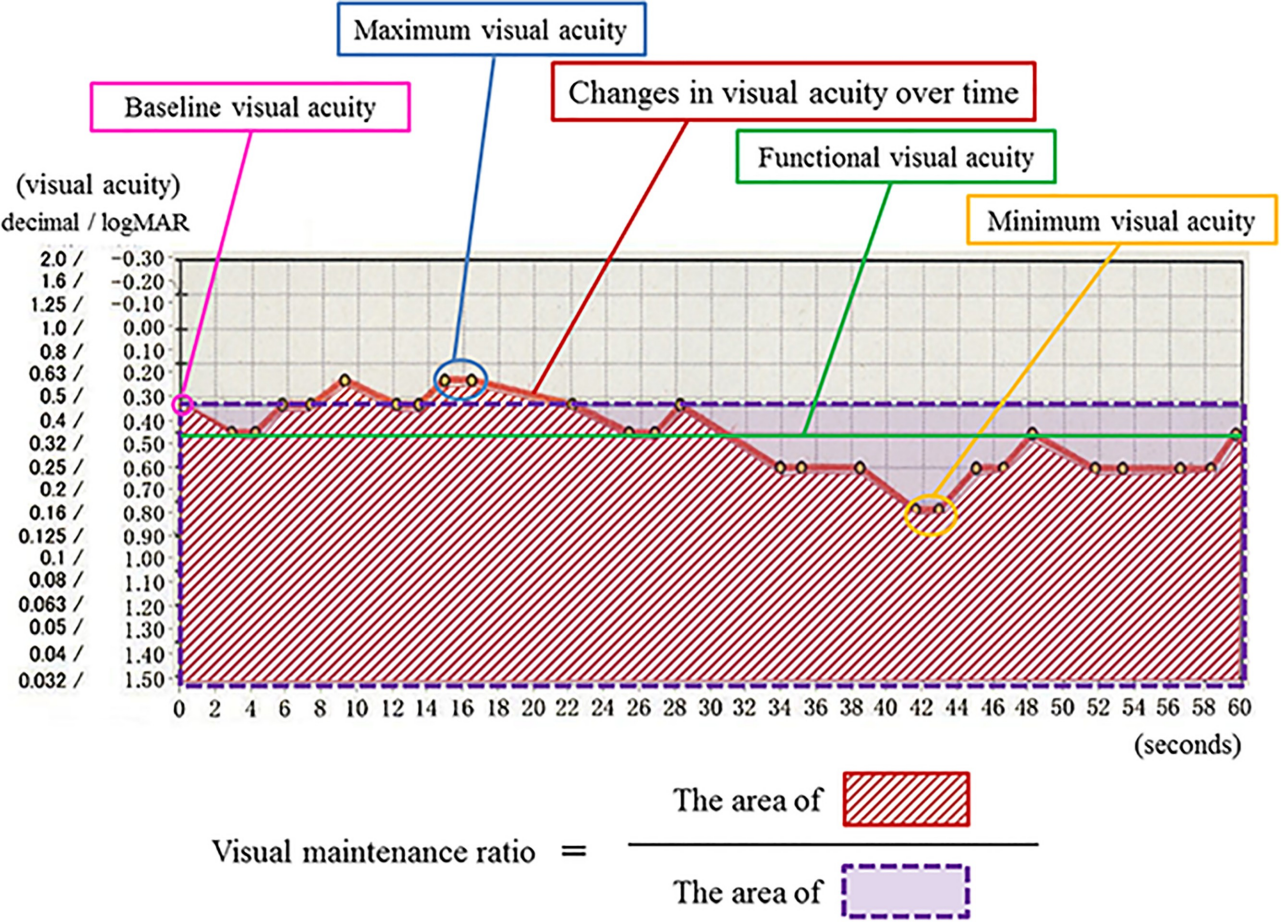
Parameters of functional visual acuity testing. Continuous red line shows sequential visual acuities measured over a 60-second measurement session. Green line denotes functional visual acuity which is calculated as the average of all visual acuity values. Pink circle represents baseline visual acuity. Visual maintenance ratio refers to area beneath time-wise change in visual acuity (red oblique line area) divided by area beneath baseline visual acuity (purple square area). Maximum and minimum visual acuities (blue and orange circles) imply the best and worst values of visual acuity over the testing period.
