# Supplementary material for: Influence of intraocular lens subsurface nanoglistenings on functional visual acuity
Source: PLoS One. 2017 Mar 22;12(3):e0173574. doi: 10.1371/journal.pone.0173574 (PMC5362055; doi:10.1371/journal.pone.0173574)
Supplement: S1 Table — (DOCX) [file pone.0173574.s001.docx]

**Table S1. Distribution of implanted IOL models in each group.**

|  | SSNG group  (eyes) | Control group  (eyes) |
| --- | --- | --- |
| MA60BM | 1 | 0 |
| MA60AC | 2 | 0 |
| MA30BA | 1 | 0 |
| SA60AT | 11 | 14 |
| SN60WF | 4 | 6 |
| Total | 19 | 20 |

IOL = intraocular lens, SSNG = subsurface nanoglistening
